# Potato Consumption and Risk of Cardiovascular Disease in a Harmonized Analysis of Seven Prospective Cohorts

**DOI:** 10.3390/nu17030451

**Published:** 2025-01-26

**Authors:** Luc Djousse, Xia Zhou, Jaewon Lim, Eunjung Kim, Howard D. Sesso, I-Min Lee, Julie E. Buring, Robyn L. McClelland, John Michael Gaziano, Lyn M. Steffen, JoAnn E. Manson

**Affiliations:** 1 Department of Medicine, Brigham and Women’s Hospital, Boston, MA 02120, USA; ekim@bwh.harvard.edu (E.K.); hsesso@bwh.harvard.edu (H.D.S.); ilee@bwh.harvard.edu (I.-M.L.); lburing@bwh.harvard.edu (J.E.B.); jmgaziano@bwh.harvard.edu (J.M.G.); jmanson@bwh.harvard.edu (J.E.M.); 2 School of Medicine, Harvard University, Boston, MA 02115, USA; 3 Department of Nutrition, Harvard T. H. Chan School of Public Health, Boston, MA 02115, USA; 4 Division of Epidemiology and Community Health, School of Public Health, University of Minnesota, Minneapolis, MN 55454, USA; zhoux062@umn.edu (X.Z.);; 5 Department of Biostatistics, University of Washington, Seattle, WA 98195-7232, USA; imjaewon@uw.edu (J.L.); rmcclell@uw.edu (R.L.M.); 6 Department of Epidemiology, Harvard T. H. Chan School of Public Health, Boston, MA 02115, USA

**Keywords:** cardiovascular disease, hypertension, epidemiology, risk factor, potato consumption

## Abstract

**Background/Objectives**: While previous study results have suggested an elevated risk of type 2 diabetes with potato consumption, limited and inconsistent results are available on the association of potato consumption with the risk of cardiovascular disease (CVD) and hypertension (HTN). We assessed the associations of (i) total potato consumption with the risk of CVD and HTN as the primary aim and (ii) fried potatoes and combined baked, boiled, and mashed potatoes with the risk of CVD and HTN as the secondary aim. **Methods**: We conducted a meta-analysis using data from seven cohorts for CVD (*n* = 110,063) and five cohorts for HTN (*n* = 67,146). Cox regression was used to estimate multivariable adjusted hazard ratios separately in each cohort and the cohort-specific results were meta-analyzed using an inverse-variance weighted method. **Results**: The mean age ranged from 25 to 72 years, 65% of the respondents were women, and the mean consumption of total potatoes ranged from 1.9 to 4.3 times per week. In the primary analysis, total potato intake was not associated with the risk of either CVD or HTN: multivariable adjusted HR (95% CI) comparing 5+ servings/week to no potato intake: 0.96 (0.89–1.04) for CVD and 1.04 (0.99–1.08) for HTN. In secondary analyses, the consumption of combined baked, boiled, and mashed potatoes was not associated with CVD or HTN; while fried potato consumption was not associated with CVD risk, there was a 10% higher risk of HTN (95% CI: 4% to 17%) comparing 1+ servings/week to no fried potato intake. **Conclusions**: While the consumption of total potato was not associated with the risk of CVD or HTN risk, a modest elevated risk of HTN but not CVD was observed only with fried potato consumption.

## 1. Introduction

Cardiovascular disease (CVD) remains one of the leading causes of death in the US and is associated with high costs [1,2,3]. Hypertension (HTN) is highly prevalent in the US and about three in four Americans will develop HTN during their lifetime [4]. Downstream consequences of HTN, including CVD, chronic kidney disease, and heart failure, contribute to high healthcare expenditure and reduced quality of life [1]. For these reasons, it is important to identify modifiable determinants of both CVD and HTN in order to reduce the burden of both conditions. Previous studies have demonstrated the critical role of healthy diet in the prevention of HTN [5,6,7,8] and CVD [9,10,11,12]. Unfortunately, the proportion of Americans with an ideal diet based on the American Heart Association classification remains low (below 3% [13]). Potatoes are widely consumed in the US and worldwide [14] and data indicated a steady increase in the prevalence of consumption worldwide. Although potatoes have a high glycemic index, they also have low energy density due to their high water content [15]. Limited and inconsistent data are available on associations of total potato consumption with the risk of CVD [16,17,18] and HTN [16]. Very few studies have evaluated associations of baked, boiled, mashed, or fried potatoes with CVD or HTN. We reported a higher incidence of CHD with 5+ cups of combined baked, boiled, and mashed potatoes per week in the Million Veteran Program [19], contrary to a recent meta-analysis of six studies, which showed no association of total potato intake with CHD [16]. Heterogeneity across studies could be partially due to a lack of adequate adjustment for potential confounding factors or an inability to differentiate methods of potato preparation (i.e., baked, boiled, or mashed, or fried potatoes). The current study addressed some of those gaps by focusing primarily on associations of total potato consumption with the incidence of CVD and HTN in seven large US cohorts. As a secondary aim, we examined the associations of combined baked, boiled, and mashed potatoes, as well as fried potato, consumption with the incidence of CVD and HTN.

## 2. Materials and Methods

**Study Population**: The current analyses are based on data collected on seven well-established prospective cohorts with data on both potato consumption and the incidence of CVD and/or HTN. Within each cohort, subjects with a prevalent outcome or missing data on potato consumption were excluded. Below is a brief description of the participating cohorts.

**The Atherosclerosis Risk in Communities (ARIC) Study** is a prospective cohort study of 15,792 participants designed to investigate the etiology of atherosclerosis and its consequences. Details on the design and methods of the ARIC study were previously published [20].

**The Coronary Artery Risk Development in Young Adults (CARDIA) Study** is a multicenter longitudinal study of 5115 subjects designed to identify risk factors of coronary artery disease. A detailed description of the CARDIA study was previously published [21].

**The COcoa Supplement and Multivitamin Outcomes Study (COSMOS)** is a randomized clinical trial designed to test the effects of multivitamins and cocoa extract supplements on cancer and CVD in 21,442 adults. The design and methods of COSMOS study were previously published [22,23].

**The Multi-Ethnic Study of Atherosclerosis (MESA)** is a study of 6814 subjects designed to study the prevalence and progression of subclinical cardiovascular disease. A detailed description of MESA was previously published [24].

**The Women’s Antioxidant Cardiovascular Study (WACS)** is a clinical trial designed to study the effects of several vitamins on cardiovascular outcomes in 8711 women. A detailed description of WACS was previously published [25].

**The Women’s Health Study (WHS)** is a clinical trial designed to assess the effects of aspirin and vitamin E on cardiovascular disease and cancer outcomes in 39,876 women. A detailed description was previously published [26].

**The Physicians’ Health Study (PHS) I** is a randomized trial designed to assess the effects of aspirin and beta-carotene on cardiovascular disease and cancer in 22,071 men. The PHS II is a trial designed to test the effects of vitamin supplements on cardiovascular disease and cancer. A detailed description of PHS I and II was previously published [27,28].

For the parent studies above, each study subject provided informed consent and each participating cohort was approved by the respective Institutional Review Boards. The current project was approved by the Institutional Review Board at Mass General Brigham (Protocol No. 2022P001795, PI: Luc Djousse).

**Assessment of potato intake**: The consumption of potatoes was assessed in each cohort using food questionnaires, including the CARDIA diet history questionnaire, Willett food frequency questionnaire, and Block questionnaire, which were previously validated [29,30,31,32]. The potato question specified the portion size, and each participant was asked to report the frequency of intake of various forms of potatoes over the previous 12 months as follows:

***ARIC***: At baseline, the participants reported their consumption of French-fried potatoes (4 oz); baked (1) or mashed potatoes (1 cup) potatoes; and potato chips or corn chips (a small bag or 1 oz).

***CARDIA***: The subjects were queried about their frequency of consumption of fried potatoes, including French fries (1 serving = 70 g); hash browns, pan fried potatoes, and potato tots (1 serving = half a cup); boiled (1 serving = half a cup); baked potatoes (1 serving = 1 medium); and other potatoes (1 serving = half a cup).

***MESA***: The participants reported their intake of French fries, fried potatoes, and hash browns; boiled/baked/mashed or other potatoes, and turnips; and potato chips and corn/tortilla chips. The subjects were provided with 3 portion sizes to choose from (small, medium, large).

***COSMOS, PHS, WACS, and WHS***: The subjects reported the frequency of consumption of French fries (serving size of 4 oz, except COSMOS, with 6 oz); baked/boiled (1) or mashed (1 cup) potatoes; and potato chips or corn chips (small bag or 1 oz).

We converted reported frequencies of potato consumption into servings per week using the midpoint for interval responses. For response categories with an open upper boundary, we multiplied the lower boundary by 1.5 [33]. For each subject, the total potato intake was obtained by summing the frequencies for fried, baked, boiled, and mashed potatoes. We kept the combined baked, boiled, and mashed potatoes as a single group. We did not have usable information on the intake of potato chips.

**Assessment of CVD and HTN**: Incident CVD was defined as myocardial infarction, stroke, or cardiovascular death adjudicated by the cohort-specific endpoint committee. HTN was defined as systolic blood pressure of 140+ mm Hg, diastolic blood pressure of 90+ mm Hg, self-reported hypertension among healthcare professionals, physician-diagnosed HTN, or treatment for HTN. While all seven cohorts participated in CVD analyses, WACS and MESA did not have data on incident HTN for inclusion.

**Covariates**: Information on age, sex, race/ethnicity, body mass index, education, smoking, alcohol intake, physical activity, dietary intake, comorbidity, and diet was obtained in each cohort at baseline.

**Statistical analysis**: Using a uniform data analysis plan, local investigators completed cohort-specific analyses and the results were meta-analyzed centrally. Covariate harmonization was implemented by participating investigators at the beginning of data analysis, inclusive of decision on whether covariates would be continuous or categorical using cohort-specific categories or quintiles. For CVD (HTN) analyses, subjects with prevalent CVD (HTN) or with missing data on potatoes were excluded. For the primary analysis, the total potato categories were <1, 1–2, >2 to 3, >3 to <5, and 5+ servings per week. For the method of potato preparation, the categories were <0.5, 0.5 to 1, >1 to 3, and >3 servings/week for combined baked, boiled, and mashed potatoes and 0, >0 to 1, and >1 servings/week for fried potatoes. Cox regressions were used to estimate hazard ratios (HRs) with 95% confidence intervals (CIs). After the unadjusted model, we fitted a multivariable model controlled for demographics and lifestyle factors. For CVD analyses, we also adjusted for prevalent hypertension and type 2 diabetes. For hypertension analyses, we also controlled for prevalent diabetes. Hazard ratios obtained from cohort-specific analyses were used for fixed-effect meta-analyses using inverse weighted variance [34]. We assessed heterogeneity using Q statistic, I-squared, and visualized using Galbraith plot in STATA SE, version 15. A two-sided p value was used with an alpha level of 0.05.

## 3. Results

A total sample of 110,063 subjects from seven cohorts was used for CVD analyses with 35.2% men and a mean age at baseline ranging from 25.1 (CARDIA) to 72.0 years (COSMOS). The mean total potato consumption ranged from 1.9 (MESA) to 4.2 (CARDIA) servings per week. Since MESA and WACS did not have data on incident HTN, only 67,146 subjects were used for HTN analyses. The baseline characteristics are shown in Table 1.

**Figure 1:**
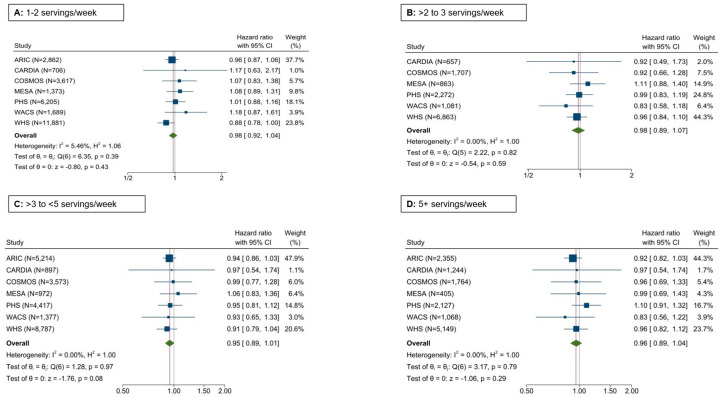
Forest plot depicting the association of the total potato intake with the incidence of CVD in seven cohorts. Cox regression adjusting for age, sex, BMI, race, education, energy intake, smoking status, alcohol intake, physical activity, prevalent hypertension and diabetes, fruits and vegetables, red/processed meat, whole grain, sugar-sweetened beverages, nuts/peanut butter, legumes, and trans fatty acids (when available).

Potato intake and CVD risk: There was no association of total potato consumption with the risk of CVD: HRs (95% CI) of 1.0 (reference), 0.98 (0.92–1.04); 0.98 (0.89–1.07); 0.95 (0.89–1.01); and 0.96 (0.89–1.04) for total potato consumption of <1, 1–2, >2 to 3, >3 to <5, and 5+ servings per week, respectively, adjusting for demographic factors, BMI, energy intake, education, physical activity, smoking, alcohol intake, prevalent hypertension and diabetes, fruit and vegetables, red/processed meat, whole grain products, sugar-sweetened beverages, nuts/peanut butter, legumes, and trans fatty acids (when available) (Figure 1). In secondary analyses, neither the consumption of combined baked, boiled, and mashed potatoes (Supplemental Figure S1) nor that of fried potatoes (Supplemental Figure S2) were associated with CVD risk.

**Figure 2:**
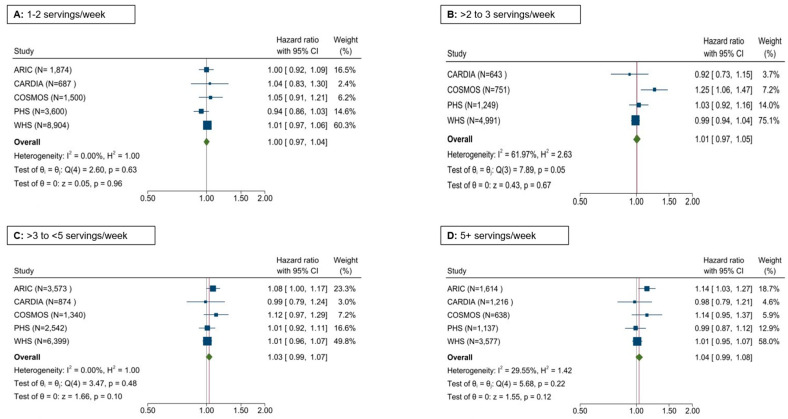
Forest plot depicting the association of total potato intake with the incidence of HTN in five cohorts; Cox regression adjusting for age, sex, BMI, race, education, energy intake, smoking status, alcohol intake, physical activity, prevalent diabetes, fruits and vegetables, red/processed meat, whole grain, sugar-sweetened beverages, nuts/peanut butter, legumes, and trans fatty acids (when available).

Potato consumption and HTN risk: Total potato consumption was not associated with the risk of HTN (Figure 2).

**Figure 3:**
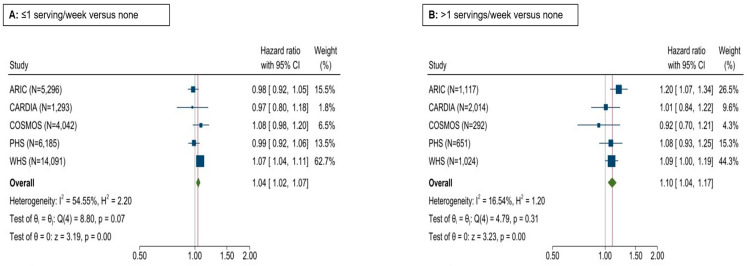
Forest plot depicting the association of fried potato intake with HTN risk in five cohorts. Cox regression adjusting for age, sex, BMI, race, education, energy intake, smoking status, alcohol intake, physical activity, prevalent diabetes, fruits and vegetables, red/processed meat, whole grain, sugar-sweetened beverages, nuts/peanut butter, legumes, and trans fatty acids (when available).

Likewise, the consumption of combined baked, boiled, and mashed potatoes was not associated with HTN risk (Supplemental Figure S3). However, the intake of fried potatoes was associated with a higher risk of HTN: HRs (95% CI) were 1.0 (reference), 1.04 (1.02–1.07), and 1.10 (1.04–1.17) for fried potato consumption of 0, >0 to 1, and >1 servings/week, respectively (Figure 3).

## 4. Discussion

In this meta-analysis of seven large prospective US cohorts, we found that total potato consumption was not associated with the risk of CVD after adjustment for potential confounding factors. Similar results were observed for combined baked, boiled, and mashed or fried potatoes. Furthermore, while the consumption of total and combined baked, boiled, and mashed potatoes was not associated with the risk of HTN, the intake of fried potatoes was associated with a higher risk of HTN.

### 4.1. Potato Consumption and Risk of CVD

A lack of an association of potato consumption with CVD risk in our study is consistent with the findings from two Swedish cohorts where total potato consumption was not associated with CVD risk [RR = 1.0 (95% CI: (0.97–1.02) per 3 servings/week increment in total potatoes] [35] and data from the Nurses’ Health Study and Health Professionals’ Follow-up study, which showed no association of potato intake with the incidence of ischemic stroke [17]. A Norwegian study also reported no association of total potato consumption with fatal myocardial infarction and CVD death after 33 years of follow-up [18]. A meta-analysis of five and six studies reported no association of total potato intake with coronary heart disease [RR = 1.02 (95% CI: 0.95–1.09)] and stroke [RR: 0.98 (95% CI: 0.88–1.08)], respectively, comparing the highest to the lowest category of total potato consumption [16].

Few studies have evaluated the association of baked, boiled, and mashed potato consumption with CVD risk. In Swedish cohorts, the consumption of boiled potatoes was not associated with the risk of CVD, myocardial infarction, stroke, or CVD death [35]. In the National Heart, Lung, and Blood Institute Family Heart Study, the consumption of baked or mashed potatoes was not associated with coronary artery calcification after adjustment for potential confounding factors [36] among 2208 US adult men and women. In contrast, data from 148,671 participants of the Million Veteran Program showed a threshold relation of combined baked, boiled, and mashed potato consumption with the risk of coronary heart disease with no association observed with an intake below 5 cups/week and 27% higher risk (95% CI: 9% to 47%) for Veterans consuming 5+ cups/week compared to <1 cup/month [19]. The reasons for inconsistent findings between US veterans and the general population are unclear and merit further investigation.

Little is known about the association of fried potato consumption with CVD risk. Our findings of no association of fried potato consumption with CVD risk are consistent with data from Larsson et al. [35], who reported no association between fried potato consumption and the risk of total CVD, myocardial infarction, or stroke in the Swedish population: compared to fried potato intake of ≤3 times per month, the multivariable adjusted hazard ratio (95% CI) for total CVD was 0.90 (0.71–1.14) in subjects consuming fried potatoes 5+ times per week.

### 4.2. Potato Consumption and Risk of HTN

Our meta-analysis of five US cohorts showed no association between total potato consumption and the incidence of HTN, and these findings are in line with a previous meta-analysis of four studies showing no association between total potato consumption and the risk of HTN [RR = 1.09 (95% CI: 0.92–1.29), *I*^2^ = 71%) when comparing the highest to the lowest category of total potato consumption] [16]. Very few studies have evaluated associations of baked, boiled, mashed, or fried potatoes with HTN risk. We showed in our data that the consumption of fried but not baked/boiled/mashed potatoes was associated with a modest elevated risk of HTN. Consistent with our findings, Schwingshackl et al. [16] reported in a previous meta-analysis that the consumption of fried [RR per 150 g/d: 1.37 (1.15–1.21)] but not baked, boiled, or mashed potatoes [RR = 1.08 (95% CI: 0.96–1.21) per 150 g/d] was associated with a higher risk of HTN. Fried potatoes may increase energy density and lead to obesity with subsequent development of HTN. It is also possible that the consumption of fried potatoes is more likely to be associated with the consumption of other food items known to raise blood pressure, including red meat, ultra-processed foods, and trans fats [37,38,39,40,41]. Added salt to fried potatoes may also contribute to a higher risk of HTN, given the blood-pressure-raising effect of salt [5,42,43,44].

### 4.3. Study Limitations and Strengths

The limitations of the current study include its observational design and the inability to completely exclude residual and/or unmeasured confounding as a partial explanation of our findings. Furthermore, potato consumption was assessed only at baseline in many cohorts and we were unable to account for potential changes in dietary habits over time. The paucity of data on the method of preparation of potatoes limits our ability to conduct robust analyses stratified by baked, boiled, and mashed alone. Since all analyzed cohorts are from the US, our results may not be generalizable to other regions of the world where food items usually consumed with potatoes, especially fried potatoes, may differ. Moreover, we lacked data on the food composition of potato-containing meals for our analyses. Since potato chips were combined with other forms of chips (i.e., tortilla or corn) in most cohorts studied, we made the decision not to include potato chips in our exposure assessment; hence, we may have underestimated the true amount of total potato consumed. Despite the above limitations, our study has several strengths, including a prospective design; a large sample size to detect small effect sizes; geographic and racial diversity of the study population; the use of harmonized and single analytical plan for cohort-specific analyses; and the availability of data on relevant potential confounding factors.

## 5. Conclusions

Our data showed that the total consumption of potato was not associated with CVD or HTN risk. In secondary analyses, baked, boiled, and mashed potato consumption was not associated with CVD or HTN, while fried potato intake was associated with a higher risk of HTN but not CVD risk.

## Figures and Tables

**Table 1:** Characteristics of seven cohorts analyzed for potato consumption and risk of CVD *.

Characteristics	Cohorts						
	ARIC (*n* = 15,106)	CARDIA (*n* = 4042)	COSMOS (*n* = 19,532)	MESA (*n* = 6215)	PHS (*n* = 19,709)	WACS (*n* = 6163)	WHS (*n* = 39,296)
Age (y)	54.2 ± 5.8	25.1 ± 3.5	72.1 ± 6.6	62.4 ± 10.3	66.3 ± 9.2	60.0 ± 8.8	54.6 ± 7.1
Sex (% women)	55.4	57.2	60.7	52.9	0	100	100
White race (%)	73.0	53.6	88.5	61.6	92.0	94.1	94.9
African American (%)	26.6	46.4	5.0	26.4	0.81	3.00	2.24
Body mass index (kg/m^2^)	27.7 ± 5.4	24.5 ± 5.0	27.6 ± 5.4	28.2 ± 5.4	25.8 ± 3.4	30.9 ± 6.8	26.0 ± 5.1
Energy intake (kcal)	1614 ± 566	2384 ± 859	1613 ± 618	1607 ± 716	1687 ± 521	1736 ± 554	1726 ± 535
Total potatoes (serv/week)	2.84 ± 2.34	4.30 ± 4.01	2.18 ± 2.33	1.85 ± 1.84	2.49 ± 2.17	2.99 ± 2.47	2.73 ± 2.17
Baked/mashed/boiled potatoes (serv/week)	2.11 ± 1.97	2.41 ± 2.99	1.71 ± 2.05	1.22 ± 1.34	1.93 ± 1.79	2.50 ± 2.21	2.29 ± 1.96
Fried potatoes (serv/week)	0.73 ± 1.08	1.89 ± 2.46	0.46 ± 0.76	0.62 ± 1.03	0.57 ± 0.93	0.48 ± 0.87	0.44 ± 0.71
Alcohol intake (drinks/week)	3.0 ± 6.6	1.78 ± 1.97	n/a	4.01 ± 8.41	n/a	n/a	n/a
Physical activity (MET-H/week or score)	2.43 ± 0.79	400.5 ± 284.9	24.0 ± 25.0	94.7 ± 97.5	n/a	n/a	14.5 ± 18.3
Fruit and vegetables (serv/week)	32.3 ± 17.9	36.4 ± 24.7	39.4 ± 28.4	30.7 ± 18.3	23.6 ± 14.4	36.8 ± 22.5	35.4 ± 21.8
Whole grain intake (serv/week)	8.48 ± 8.25	10.44 ± 10.22	6.48 ± 6.34	5.46 ± 4.99	11.8 ± 11.8	9.60 ± 8.60	10.4 ± 8.9
Red/processed meats (serv/week)	7.30 ± 5.06	30.1 ± 22.3	6.01 ± 5.58	5.47 ± 4.17	6.05 ± 5.11	7.10 ± 5.75	6.29 ± 5.04
Sugar-sweetened beverages (serv/week)	3.63 ± 6.67	11.7 ± 13.2	1.23 ± 3.50	2.75 ± 6.04	2.02 ± 3.91	2.53 ± 5.71	22.1 ± 12.2
Legumes (serv/week)	0.78 ± 1.17	1.60 ± 3.56	3.28 ± 3.94	2.78 ± 4.26	3.68 ± 3.36	3.33 ± 3.00	3.06 ± 2.75
Nuts/peanut butter (serv/week)	0.88 ± 1.85	3.57 ± 6.81	5.08 ± 6.77	1.82 ± 2.76	1.86 ± 2.80	1.27 ± 2.14	1.32 ± 2.16
Trans fat intake (g/d)	2.88 ± 1.73	n/a	0.85 ± 0.28	3.41 ± 2.28	1.72 ± 0.69	n/a	2.28 ± 1.06
Prevalent hypertension (%)	53.5	33.7	58.4	44.9	45.6	73.7	25.8
Prevalent diabetes (%)	21.6	8.7	14.2	12.5	7.0	19.2	2.85

* ARIC: Atherosclerosis Risk in Community; CARDIA: The Coronary Artery Risk Development in Young Adults Study; MESA: Multi-Ethnic Study of Atherosclerosis; n/a: Not available; PHS: Physicians’ Health Study; COSMOS: COcoa Supplement and Multivitamin Outcomes Study; WACS: The Women’s Antioxidant Cardiovascular Study; WHS: Women’s Health Study.

## Data Availability

CARDIA complies with the data-sharing requirements of the National Institutes of Health by providing limited-access data sets from various CARDIA examinations to the National Heart, Lung, and Blood Institute BioLINCC. Data from CARDIA and ARIC are available through BioLINCC.

## Supplementary Materials

The following supporting information can be downloaded at: https://www.mdpi.com/article/10.3390/nu17030451/s1, Figure S1. Forest plot depicting adjusted hazard ratios (95% confidence intervals) for CVD comparing intakes of various levels of baked, boiled, and mashed potatoes to none. Figure S2. Forest plot showing adjusted hazard ratios (95% confidence intervals) for hypertension comparing intakes of various levels of baked, boiled, and mashed potatoes to none. Figure S3. Adjusted hazard ratios (95% confidence intervals) for CVD comparing intakes of fried potatoes (>0 to ≤1 and >1 servings/week) to none.

## Abbreviations

ARIC	Atherosclerosis Risk in Communities
CARDIA	The Coronary Artery Risk Development in Young Adults Study
CI	Confidence Interval
COSMOS	COcoa Supplement and Multivitamin Outcomes Study
CVD	Cardiovascular Disease
HR	Hazard Ratio
HTN	Hypertension
MESA	Multi-Ethnic Study of Atherosclerosis
PHS	Physicians’ Health Study
RR	Relative Risk
T2D	Type 2 Diabetes
WACS	The Women’s Antioxidant Cardiovascular Study
WHS	Women’s Health Study
