# Supplementary material for: Potato Consumption and Risk of Cardiovascular Disease in a Harmonized Analysis of Seven Prospective Cohorts
Source: Nutrients. 2025 Jan 26;17(3):451. doi: 10.3390/nu17030451 (PMC11820226; doi:10.3390/nu17030451)
Supplement: Supplementary file 1 [file nutrients-17-00451-s001.zip › nutrients-3439793-supplementary.pdf]

Supplemental figures

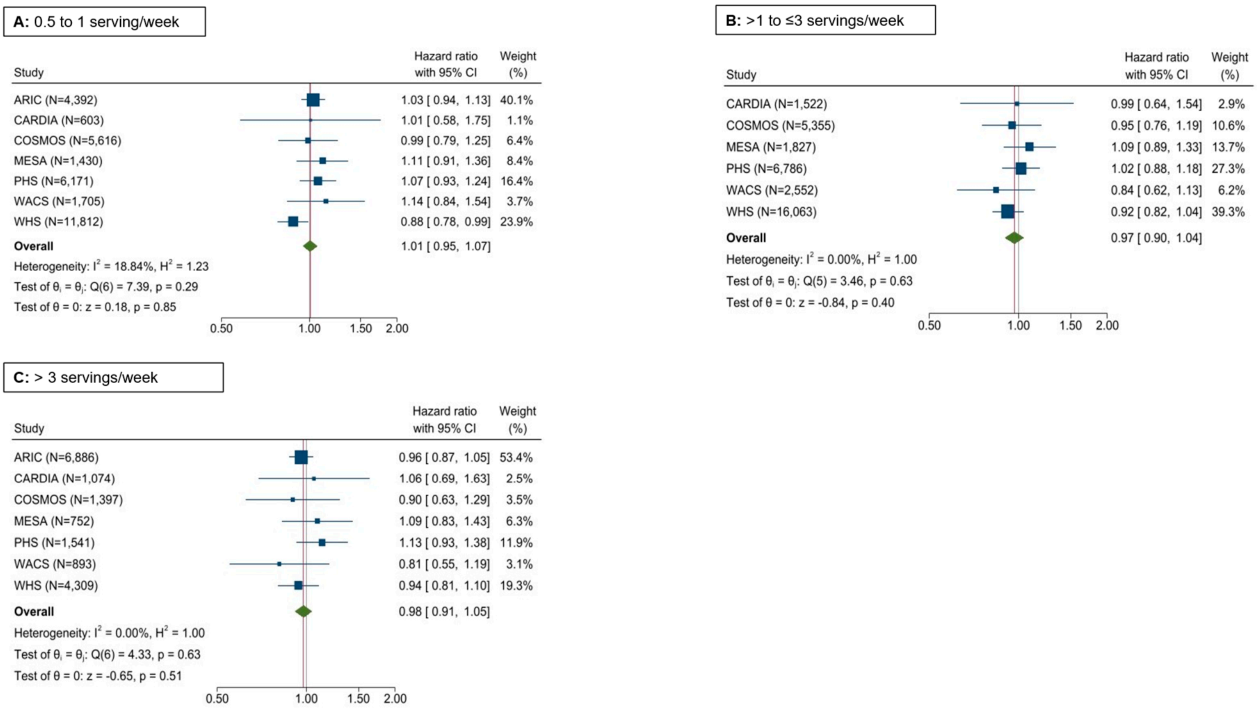

Figure S1. Forest plot depicting adjusted hazard ratios (95% confidence intervals) for CVD comparing intakes of various levels of baked, boiled, and mashed potatoes to none.

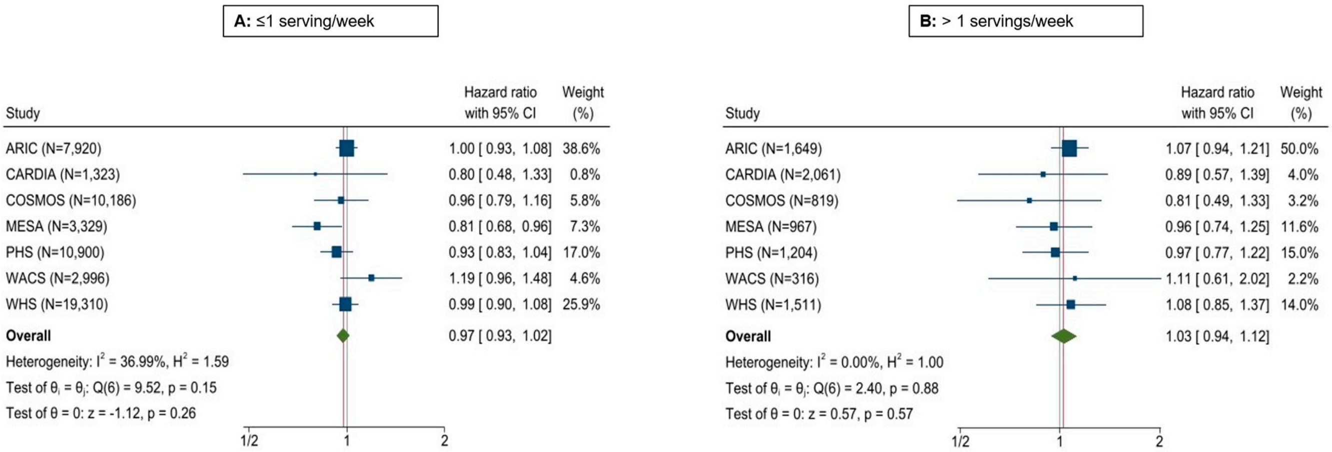

Figure S2. Forest plot showing adjusted hazard ratios (95% confidence intervals) for hypertension comparing intakes of various levels of baked, boiled, and mashed potatoes to none.

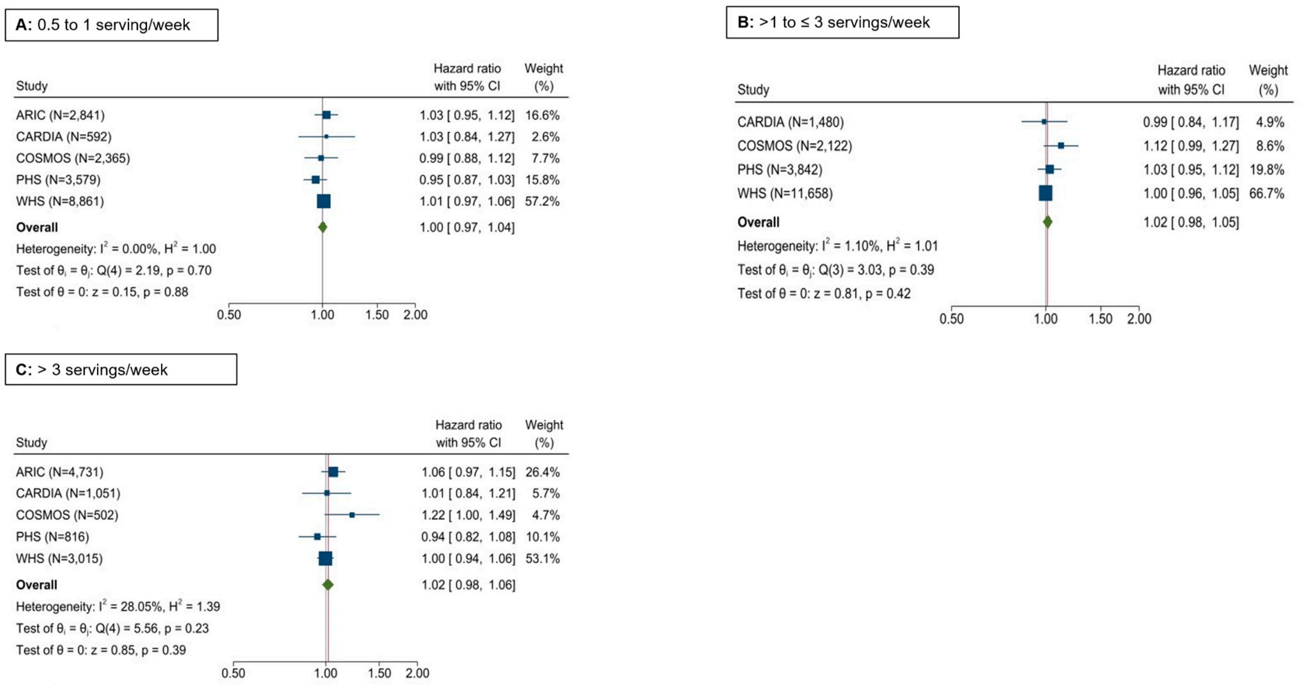

Figure S3. Adjusted hazard ratios (95% confidence intervals) for CVD comparing intakes of fried potatoes (>0 to ≤1 and >1 servings/week) to none.
